# The Clinical Spectrum of Acquired Hypomagnesemia: From Etiology to Therapeutic Approaches

**DOI:** 10.3390/biomedicines13081862

**Published:** 2025-07-31

**Authors:** Matteo Floris, Andrea Angioi, Nicola Lepori, Doloretta Piras, Gianfranca Cabiddu, Antonello Pani, Mitchell H. Rosner

**Affiliations:** 1 Nephrology, Dialysis and Transplantation Unit, ARNAS G. Brotzu, 09134 Cagliari, Italy; andrea.angioi@aob.it (A.A.); doloretta.piras@aob.it (D.P.); gianfranca.cabiddu@aob.it (G.C.); antonellopani@aob.it (A.P.); 2 Department of Medical Sciences and Public Health, University of Cagliari, 09134 Cagliari, Italy; 3 Division of Nephrology, University of Virginia Health System, Charlottesville, VA 22903, USA; mhr9r@virginia.edu

**Keywords:** magnesium, hypomagnesemia, hypokalemia, malabsorption, drug, therapy, adverse effects, proton pump inhibitors (PPIs), cancer, platinum, cetuximab, sodium-glucose cotransporter 2 inhibitors (SGLT2i)

## Abstract

Hypomagnesemia is a frequent and often underrecognized electrolyte disturbance with important clinical consequences, especially in hospitalized and critically ill patients. This multifactorial condition arises from impaired intestinal absorption, renal magnesium wasting, and the effects of various medications. Magnesium, the second most abundant intracellular cation, is crucial in enzymatic and physiological processes; its deficiency is associated with neuromuscular, cardiovascular, and metabolic complications. This narrative review focuses on the mechanisms and clinical consequences of drug-induced hypomagnesemia, highlighting the major drug classes involved such as diuretics, antibiotics, antineoplastic agents, and immunosuppressants. Management strategies include magnesium supplementation and adjunctive therapies like amiloride and SGLT2 inhibitors to reduce renal magnesium losses. Recognizing and addressing drug-induced hypomagnesemia is essential to improve patient outcomes and prevent long-term complications.

## 1. Introduction

Hypomagnesemia, clinically characterized by a serum magnesium concentration below 0.65 mmol/L, represents a multifaceted clinical issue with prevalence rates ranging from 2% in community settings to an alarming 65% in intensive care units [1,2]. Beyond genetic forms of hypomagnesemia, the etiology of this condition is multifaceted, encompassing decreased intake, diminished intestinal absorption, and excessive renal excretion [3,4,5]. Given the crucial role of magnesium in cellular processes and metabolic regulation, its deficiency can induce a spectrum of symptoms, from neuromuscular disturbances to cardiac arrhythmias. Chronic hypomagnesemia further elevates the risk for long-term metabolic conditions like diabetes, chronic kidney disease (CKD), and cardiovascular diseases [6,7,8]. A diverse array of pharmacological agents prominently feature as causative elements in both acute and chronic hypomagnesemia. Recognizing and understanding the multifactorial nature of drug-induced hypomagnesemia is vital for the timely diagnosis and efficient management of this electrolyte imbalance.

This paper intends to elucidate the complex interplay of factors affecting magnesium homeostasis, explore the various causes of acquired hypomagnesemia, detail its clinical manifestations, and propose strategic approaches for effective treatment. In examining this multifaceted issue, an extensive review of the literature from 1994 to 2024 was conducted through PubMed, focusing on terms such as magnesium, hypomagnesemia, drugs, medications, treatment, and therapy. The selection of studies for review was carefully curated based on their relevance to the topic of hypomagnesemia, with particular attention paid to those that delved into the drug-induced origins of the condition or the nuances of its treatment. Through this comprehensive examination, we aim to highlight the importance of magnesium in health and disease and provide a roadmap for clinicians in managing hypomagnesemia, emphasizing drug-induced cases.

### Search Methodology

To support this narrative review, we performed a structured literature search using PubMed, covering studies published between January 1994 and May 2054. We used combinations of the following keywords: *magnesium*, *hypomagnesemia*, *drug-induced*, *magnesium depletion*, *electrolyte disturbances*, and *magnesium supplementation*. Only articles in English were considered. We included original studies, systematic reviews, clinical trials, and relevant observational studies that addressed the pathophysiology, clinical impact, and therapeutic approaches to acquired hypomagnesemia, with particular attention to drug-induced causes. Articles were selected based on clinical relevance, mechanistic clarity, and the quality of evidence. Approximately 350 articles were initially screened, and 137 were deemed appropriate and included in the final synthesis.

## 2. Overview of Magnesium Absorption and Renal Handling

An adult’s body contains between 22 and 26 g of magnesium (Mg^2+^) [9,10]. The vast majority, over 99%, of this magnesium resides within cells, primarily in bones (accounting for 50–65%), muscles, and other soft tissues (making up 34–39%) [11]. The extracellular space contains less than 1% of the body’s total magnesium [12]. Within cells, the magnesium levels surpass those in serum. Still, due to the regulation and binding by proteins and ATP and the storage within organelles like mitochondria, the concentration of free magnesium ions remains comparable between these areas.

Due to the negative charge within cells, transporting magnesium into cells does not require energy, allowing magnesium to move passively [13]. In contrast, moving magnesium out of cells is an energy-dependent process against this charge gradient. Therefore, the movement of magnesium across cell membranes is an active transport mechanism [13].

In healthy individuals, serum magnesium levels are maintained within a narrow range of 1.6 to 2.3 mg/dL. Around 70% of magnesium in the plasma is in its ionized (free) form, crucial for various physiological functions such as nerve impulse transmission and cardiovascular system functioning [10].

**Figure 1:**
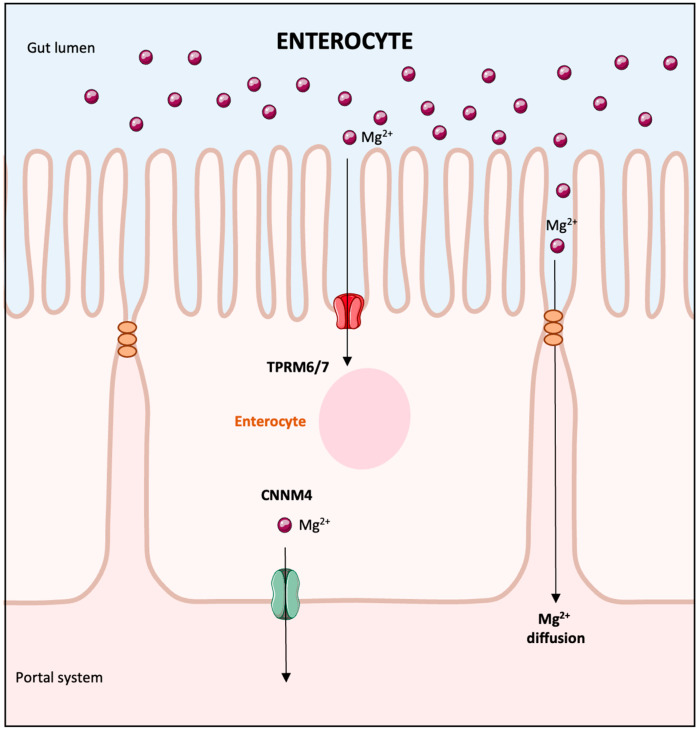
Intestinal magnesium transport. The assimilation of magnesium (Mg^2+^) in the gut involves both passive diffusion across cellular junctions and active transport through specific channels known as TRPM6/7 located within the cells lining the intestines. Once absorbed, magnesium is then transported to the bloodstream through the portal system. Proton pump inhibitors interfere with magnesium absorption by inhibiting the active transport mechanisms of the TRPM6/7 channels. The protein CNNM4 functions as a sodium-magnesium exchanger and plays a role in this process. Abbreviations: Mg^2+^: magnesium; TRPM6/7: transient receptor potential melastatin 6/7; CNNM4: sodium-magnesium exchanger cyclin M4.

Magnesium balance involves the intestines, which absorb magnesium from food and excrete part of the excess through feces; bones, which store it; and kidneys, which control the excretion through changes in urinary excretion [11]. Furthermore, although the amount of magnesium lost through sweating is usually minimal, vigorous physical activity can increase significantly [14]. The body absorbs 30–50% of the average daily intake of 370 mg, about 100 mg. With low dietary intake, the absorption efficiency can increase to 80%. Magnesium is predominantly absorbed in the small intestine, with a portion also absorbed in the large intestine [3] (Figure 1). Two distinct mechanisms facilitate magnesium absorption in the gastrointestinal tract. The first, *paracellular transport*, is a passive process driven by an electrochemical gradient, which manages the majority of absorption primarily in the small intestine [15]. The second pathway, transcellular transport, is responsible for Mg absorption in the cecum and colon and involves the proteins transient receptor potential channel melastatin (TRPM) 6 and 7 [16,17]. 

Magnesium is moved out of the intestinal cells and into the bloodstream via the portal vein, probably facilitated by cyclin and CBS domain divalent metal cation transport mediator 4 (CNNM4), which might operate as a Na^+^/Mg^2+^ antiporter exchanging sodium for magnesium ions [18]. Out of the total magnesium ingested through diet, only about a quarter to three-quarters is absorbed by the gut, with the remainder excreted in the stool [19]. Unlike other minerals, the regulation of intestinal magnesium absorption is not very precise and is primarily dictated by dietary intake. Consequently, the kidneys are believed to be dominant in maintaining magnesium homeostasis [3,11].

**Figure 2:**
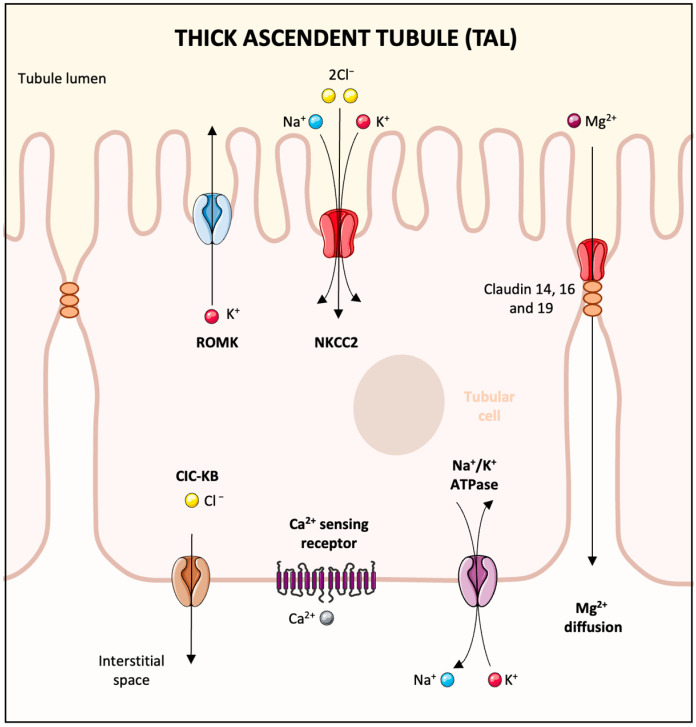
Mechanism of magnesium reabsorption in the thick ascending limb of the loop of Henle. In the TAL, the apical NKCC2 and the renal outer medullary potassium channel (ROMK) facilitate the reabsorption of cations, primarily Ca^2+^ and magnesium (Mg^2+^), through a paracellular pathway mediated by tight junction proteins (claudins 10, 14, 16, 19). Activation of the calcium-sensing receptor by high calcium levels leads to the inhibition of NKCC2 via its effects on ROMK and also suppresses passive paracellular calcium absorption through the modulation of claudins. Abbreviations: TAL: thick ascending limb of the loop of Henle; NKCC2: sodium-potassium-chloride (Na^+^-K^+^-2Cl^−^) cotransporter; ROMK: renal outer medullary potassium channel; Ca^2+^: calcium; Mg^2+^: magnesium; Na^+^: sodium; K^+^: potassium, Cl^−^: chloride; ClC-Kb: chloride channel Kb.

**Figure 3:**
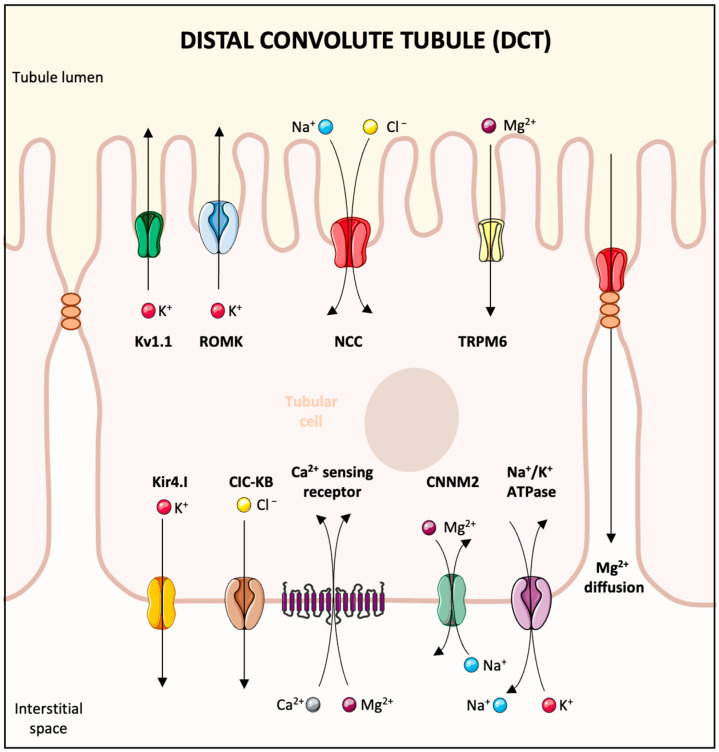
Mechanism of magnesium reabsorption in the distal convoluted tubule. In the distal convoluted tubule, the mechanism for reclaiming Mg^2+^ from the urine involves active transport through the cell membrane via the TRPM6 channels. An array of basolateral proteins, including the sodium-potassium ATPase pump, the ClC-Kb chloride channel, and the Kir4.1 potassium channel, work together to maintain a favorable gradient for Mg^2+^ entry into the cell. Furthermore, the activity of the apical ROMK and the NCC enhance the efficiency of magnesium uptake into the epithelial cells. The Mg^2+^ outlet from the cell is facilitated, in part, by the CNNM2 transporter. The modulation of magnesium transport within this tubule segment can be influenced by EGF and insulin, which upon receptor interaction, increase the TRPM6 channels’ presence and activity on the membrane, thereby augmenting Mg^2+^ reabsorption. Abbreviations: Mg^2+^: magnesium; Na^+^: sodium; K^+^: potassium; Cl^−^: chloride; TRPM6: transient receptor potential melastatin 6; ROMK: renal outer medullary potassium channel; NCC: sodium-chloride cotransporter; CNNM2: cyclin M2; EGF: epidermal growth factor.

The elimination of magnesium via the urine exhibits a daily pattern, peaking at night [20]. In a typical scenario, the kidneys’ glomeruli filter around 2400 mg of magnesium daily. About 95% of this filtered amount is reabsorbed, leading to a urinary excretion of merely 3–5%, equating to roughly 100 mg of magnesium [21]. The kidneys demonstrate flexibility in magnesium management; they can significantly reduce magnesium excretion during low magnesium availability or increase it when there is an excess intake [3]. Magnesium reabsorption within the kidney is unique compared with other ions. The principal site is not the proximal tubule, where 10–25% of Mg^2+^ is reclaimed through paracellular transport, but rather the thick ascending limb of Henle’s loop (TAL) [21] **(**Figure 2). This segment alone reabsorbs 60–70% of magnesium through paracellular transport contingent on the Na^+^ and K^+^ uptake [22]. The distal convoluted tubule (DCT) is where the finer adjustments of Mg^2+^ reabsorption are made, accounting for around 10% [22]. Transient receptor potential melastatin-like 6 (TRPM6) in the DCT is responsible for transcellular Mg^2+^ uptake from the forming urine, which relies on the voltage gradient created by the K^+^ back leak through the renal outer medullary K channel (ROMK) and K_v_1.1 potassium channels [23] (Figure 3). Magnesium transport regulation in the DCT is influenced by epidermal growth factor (EGF) and insulin, with the activation of the EGF receptor (EGFR) and insulin receptor (IR) sparking a signaling cascade through phosphoinositide 3-kinase (PI3K), protein kinase B (Akt), and Ras-related C3 botulinum toxin substrate (Rac1), leading to increased TRPM6 membrane expression and channel activity [3]. Estrogens have also been shown to augment TRPM6 expression [24].

## 3. The Magnesium Role in Physiological Processes

Magnesium is pivotal in more than 500 enzymatic processes including DNA and protein synthesis, glycolysis, and oxidative phosphorylation [25,26]. Enzymes such as adenylate cyclase and sodium-potassium-adenosine triphosphatase require magnesium to function correctly [27].

Beyond its enzymatic roles, magnesium stabilizes RNA, DNA, mitochondria, and ribosomes, contributing to the structural integrity of these critical cellular components [28]. Magnesium’s influence extends to the immune system, where variations in its levels have been linked to changes in the concentrations of several immune response mediators including interleukin-1, tumor necrosis factor-alpha, interferon-gamma, and substance P [29].

Furthermore, magnesium is integral to numerous physiological processes. It helps maintain cell membrane stability, is essential for synthesizing proteins and nucleic acids [30], regulates the tone of cardiac and smooth muscles [31], and keeps the structure of the cytoskeleton [32]. Consequently, magnesium’s involvement is crucial for ATP metabolism, muscle dynamics, neurological functions, and neurotransmitter release [33]. It also plays an essential role in regulating vascular and heart rhythms, clot formation, and bone development [34]. Magnesium also interacts with calcium, often competing for binding sites and acting as a natural counterbalance to calcium’s effects [35]. This includes its role in neurotransmitter release and cell death processes, thus inhibiting calcium-induced apoptosis [35,36].

## 4. Causes of Hypomagnesemia

The causes of acquired hypomagnesemia can be categorized into four categories: decreased intake, decreased intestinal absorption, gastrointestinal losses, and increased urinary loss. Table 1 lists common drugs associated with hypomagnesemia.

### 4.1. Decreased Magnesium Intake

The Recommended Dietary Allowance (RDA) for magnesium is 400–420 mg daily for adult men and 310–320 mg for adult women [37]. In developed countries, the average daily consumption slightly exceeds 4 mg per kilogram of body weight [38]. Low magnesium intake is often due to factors such as alcohol use disorders, the provision of total parenteral nutrition to the critically ill, and chronic fasting [34]. Hospitalized patients with alcohol use disorders commonly show low magnesium levels, a condition influenced by poor diet but also by complications like acute pancreatitis and diarrhea. In the critically ill, hypomagnesemia frequently emerges as a complication associated with higher mortality, increased incidence of sepsis, dependency on mechanical ventilation, and longer ICU stays [34].

Magnesium inadequacy is particularly problematic in specific groups, especially when the intake does not meet the RDA [39]. Those with gastrointestinal disorders like Crohn’s disease, celiac disease, or regional enteritis often face magnesium depletion due to chronic diarrhea and malabsorption [4]. Intestinal surgeries can exacerbate this issue through malabsorption and the loss of magnesium [40].

Total parenteral nutrition (TPN), often required after surgeries or when tumor obstructions prevent normal digestion, requires significant magnesium [41]. Balance studies show that patients treated with TPN retain about 0.5 mEq of Mg per gram of nitrogen, underlining the substantial magnesium needs and the risk of hypomagnesemia during TPN treatment [41].

Individuals with type 2 diabetes may suffer from magnesium depletion due to insulin resistance, which is often accompanied by increased urinary magnesium loss linked to elevated glucose levels in the kidneys [42].

For older adults, magnesium intake often decreases with age, not only because of reduced dietary intake, but also because of less efficient absorption and greater urinary excretion; moreover, chronic diseases and certain medications may affect magnesium status in this demographic [43].

### 4.2. Increased Digestive Losses

Hypomagnesemia can occur due to a variety of gastrointestinal disturbances that include vomiting and the use of nasogastric tubes for suction, enteritis, inflammatory bowel disease, the presence of intestinal and biliary fistulas, surgical resection of intestinal segments, and diarrhea [3,4,5]. Magnesium deficiency is more commonly associated with diarrhea than vomiting. This is because the secretions from the lower intestinal tract contain a much higher concentration of magnesium, up to 15 mEq/L, compared with the upper tract secretions, which contain only 1 mEq/L [44].

Cancer and its treatments can lead to chronic diarrhea through various mechanisms including secretory, osmotic, inflammatory, and dysmotility-related processes [41,45]. Neuroendocrine tumors, for instance, often cause secretory diarrhea with large-volume stools, as seen with carcinoid tumors and related serotonin syndrome leading to secondary hypomagnesemia [41].

Surgical cancer treatments can also disrupt gastrointestinal function. Cytoreductive surgeries, for instance, may result in a shortened gut or dumping syndrome, where the rapid transition of undigested nutrients into the small bowel causes osmotic diarrhea [3].

Hypomagnesemia is also commonly seen in acute pancreatitis, and its mechanisms are multifactorial. The underlying pathophysiology may be similar to that of hypocalcemia, which is often observed in this condition, where both magnesium and calcium become sequestered in necrotic fat tissue in saponified forms [46]. However, additional factors include significant gastrointestinal losses caused by vomiting, diarrhea, and nasogastric suction as well as the redistribution of magnesium from the extracellular to the intracellular space during an acute inflammatory response. Renal magnesium wasting may also occur, particularly in the context of alcohol-induced tubular injury, volume depletion, or diuretic use. Nutritional deficiencies, which are common in patients with chronic alcohol use or malnutrition, further predispose individuals to magnesium depletion. Experimental models have shown that acute pancreatitis itself can decrease the serum and tissue magnesium levels due to increased metabolic demand and systemic stress [46]. These abnormalities might go unnoticed because serum magnesium is not routinely measured in acute pancreatitis, and related symptoms are often attributed to other electrolyte imbalances or metabolic issues. Notably, hypomagnesemia frequently occurs alongside hypokalemia and hypocalcemia, both of which may be resistant to treatment unless the magnesium levels are restored.

Numerous medications known to induce severe diarrhea can lead to hypomagnesemia. This includes a range of laxatives used for constipation relief or colonoscopy prep, where even magnesium-containing products can paradoxically cause magnesium depletion if they result in substantial magnesium loss through stool [47].

Chemotherapy drugs that cause mucosal damage and shedding are linked to diarrhea and consequential magnesium loss. Up to 80% of patients on chemotherapeutic agents like capecitabine, fluorouracil, irinotecan, taxanes, platinum compounds, and targeted agents experience diarrhea, with severe cases in about 30% [45].

Antibiotics can also lead to hypomagnesemia by causing antibiotic-associated diarrhea, disrupting normal gut flora, irritating the mucosa directly, or precipitating Clostridioides Difficile colitis [48].

Colchicine, especially at higher dosages, can cause diarrhea and potential hypomagnesemia due to severe diarrheal episodes [49].

Additionally, patiromer, a calcium-exchanging, potassium-binding agent used for hyperkalemia, was found in clinical trials to cause hypomagnesemia due to impaired GI absorption in about 7% of patients, indicating that it is not entirely potassium-selective [50].

Hypomagnesemia is recognized as a common complication of proton pump inhibitors (PPIs), affecting up to 19% of users [51]. This association was established in 2006 [52] and has been supported by many case reports and observational studies. A meta-analysis of over 131,000 patients found a significant correlation between PPI use and hypomagnesemia, with a pooled adjusted odds ratio of 1.71 [51]. A systematic review of 36 cases demonstrated that the discontinuation of PPIs resulted in recovery from hypomagnesemia within four days, and rechallenge led to reoccurrence within four days. Urinary magnesium excretion is low in these patients, suggesting normal kidney function, and thus an effect of PPI use on intestinal magnesium absorption [53]. The likely mechanism involves the suppression of intestinal TRPM6 and TRPM7 channels by inhibiting the activity of the colonic H-K-ATPase, resulting in the reduced extrusion of protons into the colon [54]. Since TRPM6 activity increases at lower external pH, decreased proton secretion may reduce TRPM6 activity, which increased TRPM6 expression may compensate [55]. Proton pump inhibitors can also affect gut microbiota diversity, and thus the luminal pH, further influencing magnesium absorption [55]. Variations in individual response due to genetic factors affecting the *TRPM6* gene or alterations in intestinal pH may explain why only certain patients develop this condition after PPI use [56]. Risk factors include long-term use (more than one year), especially in the elderly and critical care patients and those affected by diabetes mellitus or chronic kidney disease, suggesting an additive effect or vulnerability to PPIs’ impact on magnesium levels [57]. Responding to these findings, the FDA released a safety announcement in March 2011 advising healthcare providers to check the serum magnesium levels in patients before starting and during long-term PPI therapy, with particular attention if they were also taking medications known to cause hypomagnesemia such as diuretics [58]. This condition can often be remedied with high-dose oral magnesium supplements and typically resolves after discontinuing PPI therapy [5].

### 4.3. Increased Urinary Loss

Urinary magnesium losses can result from an array of acquired or intrinsic mechanisms. Acquired causes might include external factors such as medications or other medical conditions that affect kidney function. Intrinsic causes are typically related to genetic conditions that affect how the kidneys handle magnesium, potentially leading to increased excretion and subsequent depletion.

## 5. Medications

### 5.1. Diuretics

Diuretics targeting the kidney’s thick ascending limb (TAL) and distal convoluted tubule (DCT) have been associated with hypomagnesemia due to their impact on renal magnesium reabsorption [59].

Loop diuretics like furosemide inhibit the NKCC2 transporter, diminishing the transepithelial voltage crucial for magnesium’s paracellular transport and increasing excretion [59]. The exact prevalence of furosemide-induced hypomagnesemia is not well-defined [60]. Animal studies show that compensatory mechanisms like upregulated TRPM6 expression in the DCT or enhanced magnesium reabsorption due to metabolic alkalosis may help mitigate the reduction in magnesium reabsorption in the TAL [61]. However, the degree of effectiveness of these mechanisms may vary among individuals [62].

Thiazide diuretics affect the NCC transporter in the DCT, and as a result, can cause magnesium loss [63]. Other effects reported in animal studies include a decrease in the expression of renal TRPM6, potentially as a secondary effect of tubular atrophy [59]. Compared with loop diuretics, thiazides are generally more associated with low magnesium levels [64]. Not all patients who use thiazides may experience hypomagnesemia. Elderly individuals and those with chronic heart failure are at a higher risk, especially if they already have low magnesium levels before starting the medication [64].

While loop and thiazide diuretics may lead to relatively mild hypomagnesemia, likely mitigated by compensatory magnesium reabsorption in the proximal tubule due to volume depletion, it is vital to monitor for magnesium depletion, particularly in patients on combination diuretic therapies, due to the potential risks involved [5]. Potassium-sparing diuretics, in contrast, tend to increase tubular magnesium reabsorption and are not typically associated with hypomagnesemia [64].

### 5.2. Antibiotics

#### 5.2.1. Aminoglycosides

Aminoglycoside antibiotics, including gentamicin, neomycin, tobramycin, and amikacin, are linked to renal magnesium loss and hypomagnesemia, with reported incidences in about one-third of patients. This condition is dose-dependent, heightens with the duration of therapy, and can persist even after discontinuation of the drug, likely as a consequence of sustained tubular damage or the drug’s residual presence in the kidneys [65].

Aminoglycosides are thought to cause hypomagnesemia by activating the calcium-sensing receptor (CaSR) in the thick ascending limb (TAL) of the loop of Henle and in the distal convoluted tubule (DCT) [65]. This activation hampers both tubular and paracellular magnesium transport, potentially leading to a Bartter-like syndrome with symptoms including renal sodium loss, hypokalemia, and hypocalcemia [66].

Furthermore, aminoglycosides have been found to reduce the expression of NKCC2, crucial for magnesium reabsorption in the TAL, and alter the renal outer medullary potassium channel (ROMK) function, further contributing to hypomagnesemia [67]. Animal studies and cellular models suggest that the body may counterbalance this effect by upregulating TRPM6 in the DCT [68].

#### 5.2.2. Amphotericin B

Amphotericin B commonly leads to hypomagnesemia, often accompanied by hypokalemia [69]. The mechanism, potentially involving a magnesium leak due to membrane disruption in the distal convoluted tubule (DCT), is not fully understood. Still, the resulting increased permeability and tubular necrosis are well-established [69]. Up to 75% of patients treated with amphotericin B may experience hypomagnesemia, which is notably more frequent with the deoxycholate form and can be exacerbated by high doses, prolonged therapy, and the concurrent use of other hypomagnesemia-inducing drugs [70,71]. Although typically reversible, hypomagnesemia can persist for weeks after discontinuing the medication [4]. Oral magnesium supplements, often given with amiloride, serve as a treatment to restore magnesium levels and mitigate this side effect [34]. It is worth noting that alongside hypomagnesemia, amphotericin B can also cause acute kidney injury and other tubular dysfunctions, such as renal tubular acidosis and nephrogenic diabetes insipidus, regardless of the glomerular filtration rate [72].

#### 5.2.3. Pentamidine

Pentamidine, an aromatic diamine compound that is effective against *Pneumocystis jirovecii* infections often found in patients with AIDS, has been associated with severe hypomagnesemia due to renal magnesium loss. While the exact mechanisms are not fully understood, pentamidine is known to disrupt ENaC activity, leading to hyperkalemia. It has been associated with tubular necrosis, which could result in DCT, contributing to hypomagnesemia. Additionally, pentamidine may induce acute pancreatitis, which in turn can cause hypomagnesemia through a process similar to that of hypocalcemia, involving the saponification of magnesium and calcium in necrotic fat tissue [73].

### 5.3. Calcineurin Inhibitors

Calcineurin inhibitors (CNIs), including cyclosporine and tacrolimus, are immunosuppressive agents widely used in organ transplantation and autoimmune disease treatment. They are notorious for causing hypomagnesemia, with tacrolimus generally inducing more severe cases than cyclosporine [74]. The development of hypomagnesemia is often attributed to renal magnesium wasting, primarily due to the downregulation of the renal expression of epidermal growth factor (EGF) and the magnesium channel TRPM6 in the distal collecting tubule [75]; there may also be a shift of magnesium into cells, further contributing to these phenomena [75]. Additional contributing factors include PPI use, post-transplantation volume expansion, metabolic acidosis, insulin resistance, deficient magnesium intake, diuretics, and decreased gastrointestinal absorption, often due to diarrhea [75]. Finally, hypomagnesemia associated with CNIs is frequently correlated with reduced kidney function, as indicated by lower serum magnesium levels associated with higher serum creatinine levels [76].

Severe neurological symptoms, such as altered mental status, seizures, and focal neurological deficits, have been reported due to CNI-induced hypomagnesemia [77]. The severity of these symptoms might be exacerbated by concurrent factors such as other medications causing hypomagnesemia, gastrointestinal losses, and comorbid illnesses [4]. Moreover, early post-transplantation hypomagnesemia has been linked to the nephrotoxic and blood pressure-raising effects of CNIs and the emergence of post-transplantation diabetes mellitus (PTDM), a significant metabolic complication post-transplantation [78]. According to some studies, in over 20% of transplant patients treated with cyclosporine, hypomagnesemia persists for many years post-surgery [75]. However, the clinical outcomes of this condition are not well-defined. Only small studies involving kidney transplant recipients with biopsy-proven cyclosporine nephrotoxicity found that hypomagnesemia was associated with a faster decline in kidney function, and this association was observed to be independent of the drug concentrations [79]. On the other hand, recently, Isakov and colleagues suggested that hypomagnesemia within the first-year post-transplant correlated with better patient and allograft survival up to ten years later [80]. This relationship held even after adjusting for clinical factors such as baseline graft function, slow graft function, calcineurin inhibitor (CNI) trough levels, and variability in CNI trough levels [80]. Given the frequency of this condition and its clinical impact on the general population or selected subpopulations (patients with diabetes, CKD, or ESRD), extensive trials are warranted to explore its clinical effect on patients treated with a kidney transplant.

### 5.4. Antineoplastic Drugs

#### 5.4.1. Platinum-Based Compounds

Chemotherapeutic agents, particularly platinum-based compounds like cisplatin, carboplatin, and oxaliplatin, are common causes of inducing renal magnesium wasting, leading to hypomagnesemia [41]. Cisplatin is most commonly associated with this side effect, and the occurrence of hypomagnesemia increases with higher drug doses [81]. This condition develops in a dose-dependent manner with cisplatin, and without adequate prophylaxis, most patients, up to 90%, may experience this side effect [81]. Cisplatin has a unique effect on inducing hypomagnesemia, which is different from oxaliplatin or carboplatin, due to its minimal binding to plasma proteins, allowing it to be unbound, freely filtered through the glomerulus, and subsequently concentrating in tubular cells via organic cation transporters [81]. This can lead to nephrotoxicity, and precisely harm magnesium reabsorption processes in the ascending limb of the loop of Henle as well as in the distal tubule [81]. The mechanism behind this renal loss of magnesium involves the drug’s cytotoxic effects on tubular cells; such damage leads to apoptosis and necrosis, which in turn results in impaired tubular magnesium reabsorption [82]. Cisplatin specifically downregulates the TRPM-6/EGF pathway, crucial for magnesium transport in the distal tubule, and may also cause a direct reduction in TRPM-6 mRNA expression or induce this effect secondary to cellular injury [83]. Gastrointestinal losses also contribute to the issue, as platinum-based treatments commonly lead to conditions such as vomiting, diarrhea, and anorexia, all of which can result in significant magnesium loss given the magnesium concentrations in the intestinal fluids [81].

This condition can persist long-term; studies have shown that even three years post-therapy, half of the patients treated with cisplatin continued to suffer from hypomagnesemia. Besides well-documented severe clinical manifestations, patients affected might manifest permanent Gitelman-like syndrome characterized by renal sodium loss, hypocalciuria, hypokalemia, and hypomagnesemia, further underlying the drug’s profound impact on renal function and electrolyte balance [84].

Carboplatin, considered less nephrotoxic than cisplatin, also leads to hypomagnesemia, albeit at a lower frequency [85]. Nonetheless, the effects of carboplatin on magnesium levels can be long-lasting and have recently been linked to reduced survival in patients with advanced ovarian cancer [86]. Proactive measures, including magnesium infusions during chemotherapy, are essential to counter the adverse effects on renal cation transporters, particularly the renal organic cation transporter 2, which facilitates cisplatin’s uptake and consequent nephrotoxicity [87]. By downregulating this transporter, magnesium can help limit the damage to the renal transport pathways and tubular cells, providing a protective effect against the nephrotoxic and hypomagnesemia-inducing properties of platinum-based chemotherapy [87].

#### 5.4.2. EGF Receptor Antagonist

Epidermal growth factor receptor (EGFR) inhibitors, such as cetuximab, panitumumab, and zalutumumab, are linked to renal magnesium wasting and hypomagnesemia [88]. Data from a large meta-analysis involving over 16,400 patients from 25 randomized controlled trials indicate that these medications can lead to hypomagnesemia in 34% of cases [89]. The incidence is significantly higher with panitumumab than cetuximab due to its longer half-life and increased affinity for the human EGFR [90]. On the contrary, zalutumumab was associated with lower rates of hypomagnesemia (4%) [91]. The underlying mechanism for this renal magnesium wasting is the inhibition of EGFR signaling. This subsequently leads to decreased TRPM-6 activity, reducing magnesium reabsorption in the distal nephron and leading to hypomagnesemia [92]. The risk of developing hypomagnesemia with these inhibitors increases with the duration of therapy, and is exacerbated by the concurrent use of medications such as platinum drugs, histamine H2 agonists, and PPIs [55]. Clinically, hypomagnesemia may manifest alongside other electrolyte imbalances, including hypokalemia and hypocalcemia, which are predominantly related to the magnesium disorder. This condition might be symptomatic and has been linked to the worsening of peripheral sensory neurotoxicity from drugs like oxaliplatin [41]. Oral magnesium supplementation can help address the deficit, but severe cases might require intravenous magnesium [93]. Notably, the discontinuation of EGFR inhibitor therapy can reverse renal magnesium wasting and hypomagnesemia. However, this recovery may vary when contrasted with the typically permanent renal magnesium wasting seen with platinum-based agents [94]. Interestingly, in the context of advanced colorectal cancer, the onset of hypomagnesemia with cetuximab or panitumumab-based chemotherapy has been correlated with delayed disease progression and extended overall survival [95]. The biological mechanism behind this correlation remains uncertain; this could be due to the impact of intracellular magnesium depletion on tumor growth or simply reflect the effective penetration of the drug into tissues [95].

#### 5.4.3. Other Antineoplastic Drugs

Other antineoplastic agents, like mTOR inhibitors (e.g., everolimus, temsirolimus), can lead to hypomagnesemia, though less often, by affecting the kidneys’ ability to handle magnesium. Additionally, ifosfamide and some alkylating agents may cause Fanconi syndrome, which results in widespread proximal tubular dysfunction and urinary magnesium loss [60].

## 6. Miscellaneous Etiologies

Hypomagnesemia can arise from various causes and has been observed in several clinical settings. Instances include post-surgical scenarios, where chelation by circulating free fatty acids occurs, and during liver transplantation due to the administration of citrate-rich blood products when the liver function is insufficient to metabolize the citrate. Furthermore, it is part of the “hungry bone” syndrome, where there is increased bone uptake of magnesium after surgical interventions for hyperparathyroidism, thyroidectomy for hyperthyroidism, or the correction of severe metabolic acidosis [96]. Moreover, hypomagnesemia is associated with diseases such as leptospirosis, partially due to urinary magnesium wasting [97].

Although medications like patiromer are primarily used for hyperkalemia, they can also result in hypomagnesemia due to their non-selective ion exchange properties [98]. Some drugs, such as beta-adrenergic and insulin, cause a cellular shift of magnesium, leading to extracellular magnesium depletion [99].

Foscarnet, a pyrophosphate analog active against cytomegalovirus complications, also causes hypomagnesemia. This effect is partly due to its role as a potent chelator of divalent cations, leading to ionized hypomagnesemia [100]. Along with hypomagnesemia, patients treated with foscarnet may experience hypocalcemia and hypokalemia, potentially as a result of magnesium disturbances [100].

In the context of bone health treatments, drugs like teriparatide have been associated with hypomagnesemia, potentially due to increased bone metabolism and transient hypercalcemia causing renal magnesium losses [101]. Bisphosphonates and denosumab are implicated due to their binding to magnesium cations [102]. Metformin is another drug implicated in hypomagnesemia, with the mechanisms believed to involve increased intracellular magnesium concentrations in erythrocytes and hepatocytes [103].

Notably, chronic alcohol abuse is a significant cause of hypomagnesemia, related to a combination of decreased intake, gastrointestinal losses, respiratory alkalosis, and excessive catecholamine release during withdrawal [104]. Alcohol-induced tubular damage and alcohol-related metabolic acidosis also play a role in causing hypomagnesemia [105].

## 7. Clinical Manifestations and Diagnosis of Magnesium Depletion

Hypomagnesemia manifestations often overlap with other biochemical abnormalities, such as hypokalemia, hypocalcemia, and metabolic alkalosis, making it challenging to attribute clinical signs specifically to magnesium deficiency [3,4,5]. Clinical features of hypomagnesemia are diverse and encompass neuromuscular, cardiovascular, and calcium metabolism disturbances.

*Neuromuscular symptoms* can range from neuromuscular hyperexcitability, including tremors, convulsions, and muscle cramps, to severe conditions like seizures and involuntary movements [106].

*Cardiovascular effects* of magnesium deficiency primarily impair Na-K-ATPase function, resulting in a spectrum of potentially fatal ventricular arrhythmias, especially during myocardial ischemia or cardiopulmonary bypass [6].

*Magnesium’s role in calcium metabolism* can lead to hypocalcemia due to hypoparathyroidism, resistance to parathyroid hormone (PTH), and the decreased synthesis of calcitriol [107]. Concurrent hypokalemia is common, resulting from shared causative conditions and increased renal potassium secretion [107]. Moreover, there are cases of normomagnesemic magnesium depletion where patients exhibit hypocalcemia that responds to magnesium therapy, suggesting possible cellular magnesium deficiency. Hypokalemia often coexists with hypomagnesemia and can be particularly resistant to treatment unless the magnesium deficit is corrected. This interrelationship stems from shared loss mechanisms and the fact that hypomagnesemia exacerbates renal potassium wasting [108].

Hypomagnesemia is also linked to a variety of *other disorders*, including nephrolithiasis [109], metabolic syndrome [110], and hypertension, both in pediatric and adult patients [6,111]. It is associated with a higher mortality in patients treated with hemodialysis [7] and the increased risk of diabetes post-transplantation [112], bone fractures [113], and the progression of diabetic kidney disease [8].

### Diagnostic Testing for Magnesium Disorders

Serum magnesium is not part of the laboratory evaluation of patients in everyday clinical practice. It is reserved for relevant clinical conditions where magnesium disorders are likely to be present or when patients present with symptoms or risks of complications that are potentially associated with magnesium disorders. Some of these circumstances may include arrhythmias, neuromuscular disturbances, diuretic use, chemotherapy exposure (such as cisplatin), malabsorption disorders, nutritional deficiency, chronic alcohol use, or unexplained hypokalemia or hypocalcemia [5]. Magnesium toxicity is suspected when exogenous magnesium is administered in the setting of decreased kidney function or in the treatment of eclampsia [114].

Measurement of the total serum magnesium concentration is the most common test for the evaluation of magnesium levels and overall body magnesium status. Ion-selective electrodes can measure the ionized fraction in serum, but the clinical utility of this value compared with the total serum concentration is uncertain [115]. One exception may be patients undergoing citrate regional anticoagulation during continuous renal replacement therapy [116]. Citrate binds magnesium and may cause reductions in the ionized fraction in the post-filter solution. Since ionized magnesium is not routinely monitored, patients undergoing this procedure may be at risk for clinically essential deficits [116].

The normal range for total serum magnesium varies somewhat between laboratories and is generally reported between 1.5 and 2.5 mg/dL. This reference range was determined from serum values obtained in healthy normal subjects participating in NHANES 1 (1971–1974) [117]. There is a suggestion that the range may need to be re-examined due to a decrease in the magnesium content in the food supply [118]. It is important to remember that a small fraction of magnesium is extracellular. Thus, the serum magnesium level is not a reliable way to assess the total body magnesium depletion [118,119]. In fact, the total body may be markedly depleted of magnesium before the serum level becomes abnormally low [120]. Some clinical clues that may signal actual magnesium depletion include persistent, unexplained hypocalcemia or hypokalemia, which is refractory to treatment with calcium or potassium [121,122].

There is some variability in the serum magnesium levels, and several factors contribute to this observation. Values tend to be higher in vegetarians when compared with those ingesting omnivorous diets [123]. Serum concentrations are higher after short periods of maximal exercise but lower after endurance exercise [14]. The serum magnesium is often lower during the third trimester of pregnancy and correlates with an increase in frequency of leg cramps [124]. Although small in magnitude, serum magnesium levels exhibit a circadian rhythm with higher values in the morning hours and lower levels in the evening [125,126]. Falsely low values for serum magnesium concentration occur with hypoalbuminemia, since approximately 30% of magnesium is bound to circulating albumin or if the sample is contaminated with potassium ethylenediaminetetraacetic acid (kEDTA) [127]. Also of note is that hemolyzed specimens can cause spuriously elevated magnesium values [128]. Despite the variability in the values noted above, measurement of the total serum magnesium concentration still provides a rapid assessment of acute changes in magnesium status and is of clinical value.

Once hypomagnesemia is confirmed, clinicians must distinguish between renal and extrarenal (gastrointestinal causes or redistribution of extracellular magnesium into the intracellular compartment) causes of magnesium wasting. Often, the clinical history provides the answer with prior use of drugs like cisplatin, active diuretic use, or chronic diarrhea. In cases where the history is not clear, a quantitative assessment of urinary magnesium excretion with a 24-h urine collection or the calculation of the fractional excretion of magnesium (FEMg) on a random urine specimen can provide insight [2]. The fractional excretion of magnesium (FEMg) is calculated as follows:

FEMg = [(UMg × PCr)/(PMg × UCr × 0.7)] × 100

In this equation, U and P are the urinary magnesium and creatinine concentrations, respectively. The 0.7-factor accounts for the 30% of magnesium that is bound to albumin and is not filtered at the glomerulus. In the setting of magnesium depletion, renal magnesium excretion should decrease from the usual value of about 3% (representing 100 mg/day) to very low levels (i.e., sometimes less than 0.5% or 12 mg/day). Therefore, an inappropriately high rate of renal magnesium excretion in the setting of hypomagnesemia confirms the diagnosis of renal magnesium wasting. Lower values suggest inadequate magnesium intake and/or gastrointestinal losses. Measurement of urinary magnesium in the hypermagnesemic patient can be used to indicate current magnesium intake.

**Figure 4:**
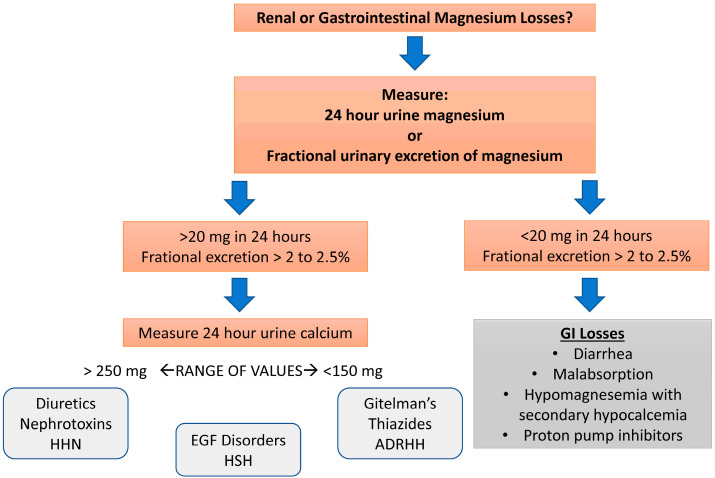
Diagnostic algorithm for hypomagnesemia. This algorithm centers on determining whether renal magnesium wasting is present or not. In the absence of renal wasting, gastrointestinal losses are likely the etiology. This algorithm also includes rare genetic etiologies of renal magnesium wasting. Abbreviations: HHN: hypomagnesemia, hypercalciuria, nephrocalcinosis; ADRHH: autosomal dominant primary hypomagnesemia with hypocalciuria; EGF: epidermal growth factor; HSH: hypomagnesemia with secondary hypocalcemia; GI: gastrointestinal.

Figure 4 depicts a diagnostic algorithm centered on the use of renal magnesium excretion to determine the etiology of magnesium wasting.

## 8. Treatment of Hypomagnesemia (Figure 5)

Whenever possible, the underlying cause of the hypomagnesemia should be corrected. The route and rate of magnesium repletion depend on the severity of the clinical manifestations. A key factor to understand in the process of magnesium repletion is the relationship between acute rises in serum magnesium and the renal excretion of magnesium [22]. Since plasma magnesium is the major regulator of magnesium reabsorption in the loop of Henle, an abrupt elevation in the plasma magnesium concentration following a bolus partially removes the stimulus for magnesium reabsorption, resulting in up to half of a bolus infusion being lost in the urine. In addition, the uptake of magnesium by cells is slow, and intracellular repletion requires sustained correction of the hypomagnesemia. For this reason, the serum magnesium may quickly increase into the normal range, only later falling to subnormal values. Thus, significant magnesium depletion requires sustained correction with repeated dosing. In patients with persistent kidney magnesium wasting disorders, the diet should be enriched with magnesium-containing foods. Such foods include green leafy vegetables, legumes such as beans and peas, nuts and seeds, and fiber-rich whole grains.

**Figure 5:**
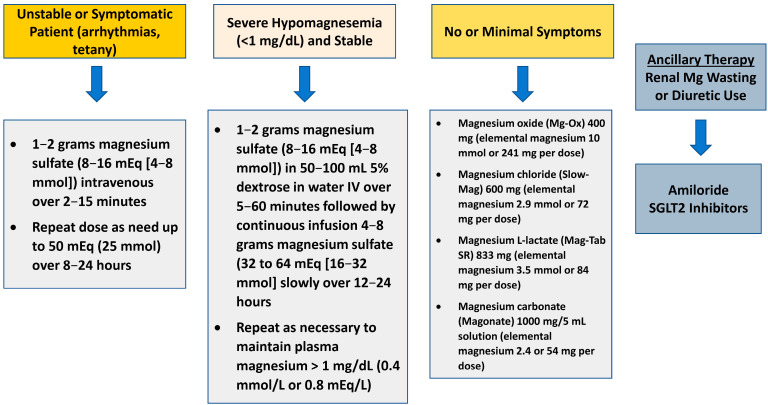
Treatment of hypomagnesemia. This algorithm stratifies patients based upon whether patients have symptoms or severe or mild hypomagnesemia. Intravenous replacement is warranted when symptoms are present and or patients present with magnesium levels < 1 mg mg/dL. In milder cases, oral therapy with a variety of agents can be utilized. Abbreviations: Mg: magnesium.

### 8.1. Severe Hypomagnesemia

If hypomagnesemia is severe (<1 mg/dL) or accompanied by symptoms such as cardiac arrhythmias, neuromuscular irritability (tetany), or seizures, parenteral magnesium therapy should be administered. Magnesium sulfate 1–2 g (8–16 mEq) can be given intravenously over 15 min. A continuous infusion should be given after the initial bolus, for example, with magnesium sulfate 4–8 g/24 h (32–64 mEq). In asymptomatic patients, this dose can be repeated as necessary to maintain the plasma magnesium level > 1 mg/dL. Patients receiving aggressive intravenous therapy require continuous cardiac monitoring along with frequent measurements of the serum magnesium (every 4–6 h). Magnesium repletion should continue for at least 1–2 days after serum magnesium normalizes because the added extracellular magnesium equilibrates slowly with the intracellular compartment. Patients with continued magnesium losses will require continued replacement. Patients should have their plasma potassium concentration monitored, since the excretion of the sulfate anion (with magnesium sulfate) can increase luminal electronegativity in the distal nephron and secondarily increase potassium excretion. Of note, symptomatic patients with severe hypomagnesemia who have reduced kidney function are at risk for iatrogenic hypermagnesemia when receiving intravenous therapy. The frequent measurement of plasma levels, along with monitoring for signs of hypermagnesemia (decreased tendon reflexes, facial flushing, and evidence of atrial-ventricular conduction block), is required in these patients. A 25–50% reduction in intravenous dosing is prudent in patients with an eGFR of <30 mL/min.

If the underlying cause of the hypomagnesemia persists once the acute emergency has been corrected, oral magnesium replacement may be necessary. Of note, patients with large amounts of gastrointestinal fluid losses (high-output ostomies) present a challenge, and some have reported efficacy with the use of intermittent subcutaneous dosing of magnesium sulfate [129].

### 8.2. Mild Hypomagnesemia

Given that significant wasting of magnesium occurs in the setting of rapid parenteral magnesium administration, treatment with oral magnesium salts is the more efficient way to replenish magnesium stores in patients who are asymptomatic or who require maintenance therapy due to chronic magnesium losses. The slower rise in the serum magnesium level that results from oral therapy provides a more favorable gradient for renal magnesium reabsorption since magnesium is slowly absorbed, limiting the rise in blood concentration. Sustained release preparations are preferable. Approximately one-third of the administered dose is absorbed with these preparations in the absence of intestinal malabsorption. There are several such preparations currently available such as Slow-Mag and Mag Delay containing magnesium chloride, and Mag-Tab SR containing magnesium lactate. These orally administered magnesium preparations are given in divided doses to decrease their cathartic effect. Six to eight tablets (30–56 mEq [15–28 mmol]) in divided doses is a reasonable starting dose for patients with severe magnesium depletion. In patients with mild, asymptomatic disease, 2–4 tablets (10–28 mEq [5–14 mmol]) daily is a reasonable starting point. Magnesium oxide may also be used, but is more rapidly absorbed, necessitating higher doses than sustained release preparations. Diarrhea is a frequent adverse effect associated with magnesium oxide due to the need for higher doses.

The duration of oral magnesium therapy depends on the underlying cause and clinical situation. In temporary conditions, such as acute gastrointestinal losses or short-term diuretic use, supplementation is usually continued for 1–2 weeks after serum magnesium is corrected to ensure intracellular replenishment. In long-term conditions, like prolonged use of proton pump inhibitors, chronic diarrhea, inherited renal magnesium loss, or chemotherapy-related hypomagnesemia, extended or even indefinite treatment may be needed. Once treatment stops, the serum magnesium levels may stay stable in patients without ongoing losses, but recurrence is common in those with persistent risk factors. Follow-up includes clinical assessment and serum magnesium measurement 1–2 weeks after stopping therapy, and periodically afterward (every 3–6 months) for patients with chronic issues. Patients on long-term treatment should be regularly evaluated for gastrointestinal tolerance, serum magnesium levels, and the possible need for dose adjustments based on kidney function and current medications.

Although magnesium supplementation is generally safe, using it without supervision, especially in people with impaired kidney function, may cause adverse effects. Patients should be warned against self-prescribing magnesium supplements without medical guidance.

If renal magnesium wasting persists despite high-dose oral magnesium replacement (as in the inherited magnesium wasting disorders, cisplatin toxicity, or other etiologies), adding potassium-sparing diuretics such as amiloride may be beneficial. These drugs decrease magnesium excretion by increasing its reabsorption in the convoluted collecting tubule. In addition, sodium-glucose cotransporter 2 inhibitors (SGLT2i) can also be used to reduce urinary magnesium excretion.

### 8.3. Drugs to Limit Kidney Magnesium Wasting

#### 8.3.1. Amiloride

Amiloride has the ability to decrease renal magnesium losses and thus has been utilized to treat certain cases of hypomagnesemia [130]. Amiloride is especially useful for diuretic-induced hypomagnesemia, cisplatin-associated magnesium wasting, and to lower urinary magnesium excretion in patients with Gitelman’s and Bartter’s syndromes. Amiloride blocks sodium transport by the epithelial sodium channel (ENaC) in the distal nephron, which hyperpolarizes the cell membrane, providing an electrical driving force favoring magnesium uptake into the cell through transient receptor potential cation channels 6 and 7 [131]. In animal studies, a dose–response relationship is seen for the effects of amiloride to reduce the fractional excretion of magnesium during furosemide-induced diuresis [132]. However, the effects of amiloride on magnesium excretion are less than its effects on potassium excretion [132].

#### 8.3.2. Sodium-Glucose Cotransporter 2 Inhibitors (SGLT2i)

Unexpectantly, clinical trials in diabetics with SGLT2i have demonstrated that the use of these drugs was associated with a rise in the plasma magnesium level by approximately 0.08–0.2 mEq/L [132]. Based upon this and other similar findings, SGLT2i have been utilized to minimize urinary magnesium wasting in a small number of patients with refractory hypomagnesemia [133]. An unproven hypothesis that has followed this observation is that the increase in serum magnesium associated with SGLT2i therapy may contribute to cardiovascular benefits specifically by decreasing the risk of cardiac arrhythmias [134]. The mechanism of the magnesium-sparing effects likely involve several mechanisms: (1) falls in insulin levels and increases in glucagon levels that may affect the tubular transport of magnesium as well as the extracellular redistribution of magnesium [135]; (2) changes in serum phosphate levels that impact changes on parathyroid hormone, fibroblast growth factor-23, and vitamin D levels that lead to changes in renal magnesium handling [136]; and (3) through change in the urinary flow rate that through changes in fluid-mediated shear forces changes tubular magnesium handling [137]. There is a limited amount of clinical experience with the use of SGLT2i in decreasing renal magnesium wasting but these drugs may offer an additional tool to treat hypomagnesemia.

### 8.4. Nutritional Management of Hypomagnesemia

Dietary correction is key in preventing and managing mild hypomagnesemia. Magnesium-rich foods include green leafy vegetables (e.g., spinach, Swiss chard), legumes (such as beans and lentils), whole grains, nuts (particularly almonds and cashews), seeds (like pumpkin and chia), and dark chocolate. Mineral water with high magnesium levels may also help. For patients with mild deficiency or as a maintenance approach after repletion, dietary counseling should be promoted to ensure sufficient intake. When dietary intake alone is insufficient, especially in malabsorption or high losses, supplementation is still needed. Monitoring should involve dietary recall and regular serum magnesium checks [26].

## 9. Research Agenda

Future research on hypomagnesemia should prioritize several areas to bridge existing knowledge gaps. First, advanced diagnostic tools must be developed for the early detection of magnesium deficiency, potentially through enhanced serum and intracellular magnesium testing methods. Additionally, studies aimed at understanding the cellular and molecular regulation of magnesium could inform targeted therapeutic interventions.

There is also a pressing need for clinical trials to test the effectiveness and safety of both new and existing magnesium supplementation approaches, especially for high-risk groups such as patients with chronic illnesses or those on magnesium-depleting medications. Investigating genetic factors that affect susceptibility to hypomagnesemia may further personalize treatment strategies.

Moreover, given magnesium’s role in multiple health conditions, interdisciplinary research involving cardiology, nephrology, endocrinology, and nutrition is essential. This approach could improve our understanding of magnesium’s systemic impacts and provide comprehensive management strategies for those affected by or at risk for hypomagnesemia.

## 10. Conclusions

In conclusion, hypomagnesemia is a critical and often overlooked electrolyte imbalance with significant clinical implications. Its complex pathophysiology requires vigilant monitoring and tailored management strategies. Understanding the nuances of magnesium metabolism and the mechanisms by which various medications influence it is crucial for the prevention and treatment of this condition. Future research and clinical focus should emphasize early detection, the refinement of treatment protocols, and patient education to mitigate the risks associated with chronic hypomagnesemia and its potential impact on long-term health outcomes.

## Figures and Tables

**Table 1:** Mechanisms behind drug-induced hypomagnesemia.

Mechanisms of Drug-Induced Hypomagnesemia
**Intracellular shift of magnesium**
Insulin therapy
Beta-agonists: Epinephrine, Salbutamol, Terbutaline, Rimiterol
Xanthines: Theophylline
Correction of metabolic acidosis with alkali therapy
Metformin
**Gastrointestinal loss of Magnesium**
Laxative abuse
Antibiotics, antineoplastic agents
Proton pump inhibitors
Colchicine
Patiromer
Chemotherapeutic agents causing intestinal mucosal injury
**Increased urinary Magnesium excretion**
Antineoplastics: Carboplatin, Cisplatin
Monoclonal antibody EGFR inhibitors: Cetuximab, Panitumumab
mTOR inhibitors
Calcineurin inhibitors: Cyclosporine, Tacrolimus
Aminoglycosides
Amphotericin B
Diuretics: Thiazides, Furosemide
Digoxin
**Miscellaneous**
Alcohol
Massive transfusions
Teriparatide
Bisphosphonates
Denosumab

## Data Availability

No new data were created or analyzed in this study. Data sharing is not applicable to this article.
